# Landscape of lipidomics in cardiovascular medicine from 2012 to 2021: A systematic bibliometric analysis and literature review

**DOI:** 10.1097/MD.0000000000032599

**Published:** 2022-12-30

**Authors:** Wenting Wang, Lei Song

**Affiliations:** a Department of Cardiovascular Disease, Affiliated Hangzhou Chest Hospital, Zhejiang University School of Medicine, Hangzhou, China; b Beijing University of Chinese Medicine, Beijing, China.

**Keywords:** bibliometric analysis, cardiovascular diseases, lipidomics, lipids, metabolomics

## Abstract

Lipidomics has shaped our knowledge of how lipids play a central role in cardiovascular diseases (CVD), whereas there is a lack of a summary of existing research findings. This study performed a bibliometric analysis of lipidomics research in cardiovascular medicine to reveal the core countries, institutions, key researchers, important references, major journals, research hotspots and frontiers in this field. From 2012 to 2021, a total of 761 articles were obtained from the Web of Science Core Collection database. There is a steady increase of publications yearly. The United States and China are on the top of the list regarding article output. The institutions with the most publications were the Baker Heart and Diabetes Institute, the Chinese Academy of Sciences and Harvard Medical School. Peter J Meikle was both the most published and most co-cited author. The major journal in this field is Journal of lipid research. Keyword co-occurrence analysis indicated that coronary heart disease, mass spectrometry, risk, fatty acid, and insulin resistance have become hot topics in this field and keyword burst detection suggests that metabolomics, activation, liver, low density lipoprotein are the frontiers of research in recent years. Collectively, lipidomics in CVD is still in its infancy with a steady increase yearly. More in-depth studies in this area are warranted in the future.

## 1. Introduction

Cardiovascular diseases (CVDs) are the leading cause of deaths globally. Although guideline-based management has improved the survival and quality of life of patients with CVD, the incidence of CVD continues to increase.^[1,2]^ Dysregulation of lipid metabolism plays a crucial role in the pathophysiology of CVD.^[3]^ Routine clinical lipidology predominantly focuses on lipid classifications, such as total triglycerides and low-density lipoprotein cholesterol rather than on a more detailed analysis of lipid entities.^[4,5]^ Notably, the evolving technology of lipidomics has shed light on a deeper understanding of perturbed lipid homeostasis in the pathophysiology of various CVD conditions, including atherosclerosis,^[6]^ coronary artery disease (CAD),^[7]^ hypertension,^[8]^ and heart failure.^[9]^

Lipidomics, a subset of metabolomics, involves the study of lipidomes using analytical chemistry technology. Nuclear magnetic resonance spectroscopy and mass spectrometry are the main analytical approaches to lipidomic analysis.^[10–12]^ Rapid advances in analytical techniques^[12,13]^ have enabled the identification and quantification of hundreds of lipid species across multiple pathways and networks under different physiological/pathological conditions.^[14,15]^ In the past decade, lipidomic analysis has been intensively applied in the field of CVDs,^[16,17]^ and series of studies are being conducted worldwide. A systematic study of lipidome could help in identifying lipids that can serve as diagnostic biomarkers and therapeutic targets in cardiovascular medicine.^[18]^

Bibliometrics is a research method that uses mathematical and statistical methods to qualitatively or quantitatively analyze a specific knowledge system.^[19]^ It has been widely used in many fields such as medicine and sociology.^[20–22]^ Based on bibliometric methods, various visual analysis tools that can be used to visualize development trends and research hotspots in a specific field have been developed. Among these tools, the CiteSpace software, which was developed by Professor Chaomei Chen of Drexel University based on the Java programming language, is widely used.^[23]^ This software can perform cooperation network analysis, co-occurrence analysis, and co-citation analysis to discover the development trends, research hotspots, and frontiers of a certain field.

Based on a literature search, we found no bibliometric analysis studies on the application of lipidomics in CVDs. Therefore, we conducted this study with the aim of summarizing and analyzing the developmental trends and research hotspots in this field over the past 10 years to help future researchers to quickly grasp the development overview of this field and to provide references for future studies.

## 2. Methods

### 2.1. Data acquisition and search strategy

All relevant literature was retrieved and exported from the Web of Science Core Collection (WoSCC) database with the search formulas Topic=(“lipidomic*” OR “lipidome*”) and Topic=(“cardiovascular” OR “heart”). The preliminary search yielded 892 records, and a total of 761 of these records were included in the data analysis set based on the results refinement function in the WoSCC database, which was used with the following settings: language, English; document types, original and review articles; and publication dates, from January 1, 2012 to December 31, 2021. The 761 records were selected as “full record with cited references,” exported into “plain text file format,” and renamed with the “download*. txt” convention to ensure that they were read correctly in the CiteSpace software.

### 2.2. Data analysis

Microsoft Excel 2019 (Microsoft Corporation, Redmond, WA) was used to prepare graphs showing the number of publications per year. The CiteSpace software was used to perform country, institution, and author cooperation network analyses; journal co-citation analysis; reference co-citation analysis; and keyword co-occurrence, cluster, and burst analyses.

The size of the nodes was positively correlated with the frequency of co-occurrence or co-citation of the analyzed items. The lines between the nodes indicated a co-occurrence or co-citation relationship, and the thickness of the lines indicated the strength of the relationship between the items. The colors of the rings and lines around a node indicated the year in which the item or relationship was first observed in the literature. The purple circles around certain nodes indicated betweenness centrality (BC), which shows the importance of a node within the entire network. Nodes with BC >0.1 were marked with purple rings. The thickness of the purple ring was proportional to the BC value.

## 3. Results

### 3.1. Publications trends and type

**Figure 1.**
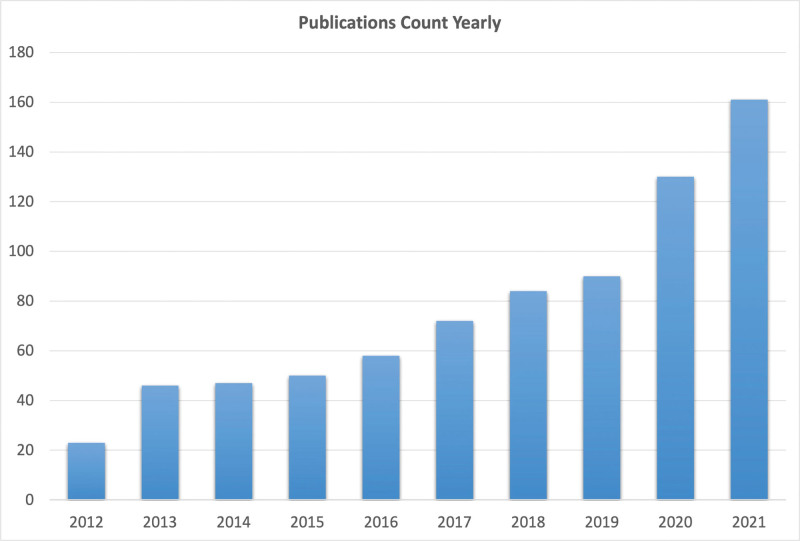
Annual trends in publications.

As shown in Figure 1, the number of publications on lipidomics in CVDs in the year 2012 was approximately 20, indicating that the field was still in its infancy and showed a steady upward trend after 2012. In 2021, the number of publications exceeded 160, an 8-fold increase compared with that in 2012, suggesting that this field is gradually receiving attention from researchers within this decade. Articles accounted for 83.05% of the published literature, indicating that the field is dominated by original articles, and summary analysis of publications are relatively lacking, indicating the significance of this study.

### 3.2. Country and institution cooperation network analyses

**Figure 2.**
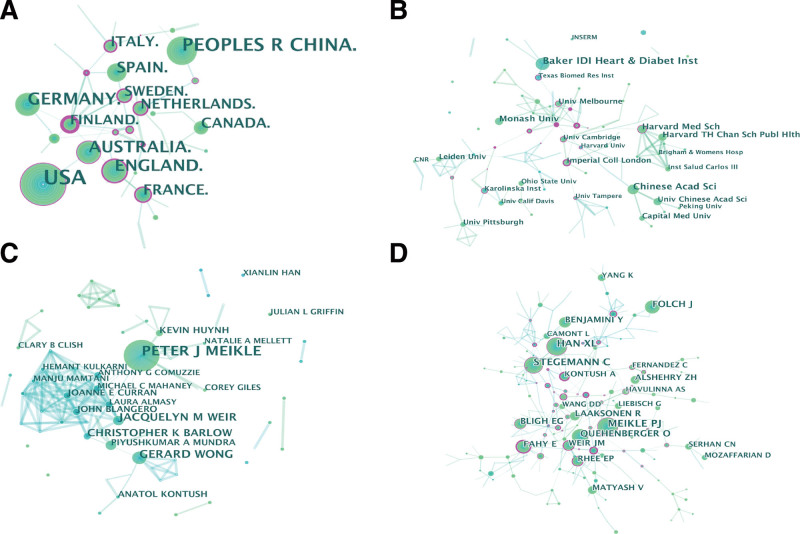
Collaborative network analysis. (A) The country cooperation network. (B) The institution cooperation network. (C) Author co-occurrence analysis network. (D) Author co-citation analysis network.

The USA had the highest number of publications in this field (269), followed by China (128), Germany (83), the UK (78), and Australia (73) (see Table S1, Supplemental Digital Content, http://links.lww.com/MD/I273), which shows the 10 countries and institutions with the highest volume of collaborative publications. The 5 most productive countries were from North America, Asia, Europe, and Australia, indicating that this field is being widely pursued by researchers worldwide. Sixty percent of the top 10 countries was identified in Europe and 20% in North America, indicating that researchers from Europe and North America are fronting developments in the field. The number of articles published from the USA (the 1st ranked country) is about twice as many as those from China, (the 2nd ranked) and about thrice as many as those from Germany (3rd ranked). Only the USA has a BC value >0.1, indicating that the USA dominates developments in this field. In addition, it is noteworthy that the UK, Australia, France, Italy, and the Netherlands have BC values >0.1 each, which are shown as nodes with purple outer circles in Figure 2A, suggesting that these countries are key nodes in the overall international cooperation network and play an important connecting role in the cooperation with other countries. The institutions with the highest number of publications were the Baker Heart and Diabetes Institute in Australia (28), the Chinese Academy of Sciences in China (21), and the Harvard Medical School in the USA (16) (Fig. 2B). Furthermore, the top 10 institutions with the highest publications are the top medical or research institutions in their respective countries, revealing that this field is receiving attention from researchers in top research institutions.

### 3.3. Authors and co-citation analyses

The top 10 authors in terms of number of publications and co-citation frequency in the field during the last 10 years are shown in (Table S2, Supplemental Digital Content, http://links.lww.com/MD/I274) which shows the top 10 authors that emerged from the co-occurrence and co-citation analyses. Among these authors, Peter J Meikle had the highest output (47), followed by Gerard Wong (18) and Jacquelyn M Weir (17). It is worth noting that Peter J Meikle ranked first in both the number of articles and co-citation frequency, suggesting that his research has contributed significantly to developments in the field (Fig. 2C). In addition, although Xianlin Han and Christin Stegeman are not among the top 10 authors in terms of the number of publications, they are ranked 2nd and 3rd, respectively in terms of co-citation frequency, with BC values >0.1, indicating that their research findings are widely cited by other researchers and are key nodes in the entire co-citation network (Fig. 2D).

### 3.4. Journals and co-cited journals analyses

The top 5 journals with the highest number of publications in this field are *Journal of Lipid Research* (30), *Atherosclerosis* (21), *Metabolites* (21), *PLoS One* (21), and *Journal of Proteome Research* (19) (see Table S3, Supplemental Digital Content, http://links.lww.com/MD/I275), which shows the top 10 journals and co-cited journals. Among these journals, *Journal of Lipid Research* (535) had the highest co-citation frequency, indicating that the papers published in this journal play an important role in promoting developments in this field. *PLoS One* (477) and *Circulation* (450) were the 2nd and 3rd most co-cited journals, respectively. Ninety percent of the top 10 journals in terms of the number of publications are in Q1 or Q2, and all the top 10 journals in terms of co-citation frequency were located in Q1 or Q2, indicating that all these journals have high academic reputation in this field.

### 3.5. Reference co-citation analysis

Co-citation analysis is a research method used to measure the degree of the relationship between articles, indicating that 2 or more articles that have been simultaneously cited by 1 or more papers have a co-citation relationship. Table 1 shows the 10 most co-cited articles, of which *Lipidomics profiling and risk of cardiovascular disease in the prospective population-based Bruneck study* ranks first (59). All these 10 articles were in Q1 or Q2, with an average impact factor of 15.9464.

**Table 1:** The top 10 co-cited references.

Frequency	Centrality	Title	Author	Year	Source	IF	JCR
59	0.19	Lipidomics profiling and risk of cardiovascular disease in the prospective population-based Bruneck study	Christin Stegemann, et, al	2014	*Circulation*	26.690	Q1
58	0.15	Plasma ceramides predict cardiovascular death in patients with stable coronary artery disease and acute coronary syndromes beyond LDL-cholesterol	Reijo Laaksonen, et, al	2016	*European Heart Journal*	29.983	Q1
46	0.11	Plasma lipidomic profiles improve on traditional risk factors for the prediction of cardiovascular events in type 2 diabetes mellitus	Zahir H Alshehry, et, al	2016	*Circulation*	26.690	Q1
39	0.19	Plasma lipid profiling in a large population-based cohort	Jacquelyn M Weir, et, al	2013	*Journal of Lipid Research*	5.922	Q1
34	0.08	Plasma lipidomic analysis of stable and unstable coronary artery disease	Peter J Meikle, et, al	2011	*Arteriosclerosis Thrombosis and Vascular Biology*	8.313	Q1
34	0.29	Plasma lipid profiling shows similar associations with prediabetes and type 2 diabetes	Peter J Meikle, et, al	2013	*PLoS One*	3.240	Q2
32	0.43	Circulating ceramides predict cardiovascular outcomes in the population-based FINRISK 2002 cohort	Aki S Havulinna, et, al	2016	*Arteriosclerosis Thrombosis and Vascular Biology*	8.313	Q1
27	0.01	Plasma ceramides, Mediterranean diet, and incident cardiovascular disease in the PREDIMED trial (Prevención con Dieta Mediterránea)	Dong D Wang, et, al.	2017	*Circulation*	26.690	Q1
26	0.04	Lipidomics: potential role in risk prediction and therapeutic monitoring for diabetes and cardiovascular disease	Peter J Meikle, et, al	2014	*Pharmacology & Therapeutics*	12.310	Q1
25	0.4	Small, dense high-density lipoprotein-3 particles are enriched in negatively charged phospholipids: relevance to cellular cholesterol efflux, antioxidative, antithrombotic, anti-inflammatory, and antiapoptotic functionalities	Laurent Camont, et, al	2013	*Arteriosclerosis Thrombosis and Vascular Biology*	8.313	Q1

IF = impact factor, JCR = journal citation reports, LDL = low-density lipoprotein.

### 3.6. Keywords co-occurrence and cluster analyses

Keywords are the distillation of the core content of an article; therefore, the analysis of keywords can be used to understand research hotspots in a certain research area. Apart from lipidomics and CVDs, coronary heart disease, mass spectrometry, risk, fatty acids, insulin resistance, oxidative stress, and plasma were the keywords with a high co-occurrence frequency, all of which exceeded 60 occurrences (see Table S4, Supplemental Digital Content, http://links.lww.com/MD/I276), which shows the top 20 keywords. The top 20 keywords can generally be grouped into the following 4 major categories: disease (coronary heart disease, insulin resistance, metabolic syndrome, atherosclerosis, myocardial infarction, and obesity), technical means (mass spectrometry, plasma, and shotgun lipidomics), pathological process (fatty acid, oxidative stress, inflammation, and cholesterol), and significance (risk factor and identification).

**Figure 3.**
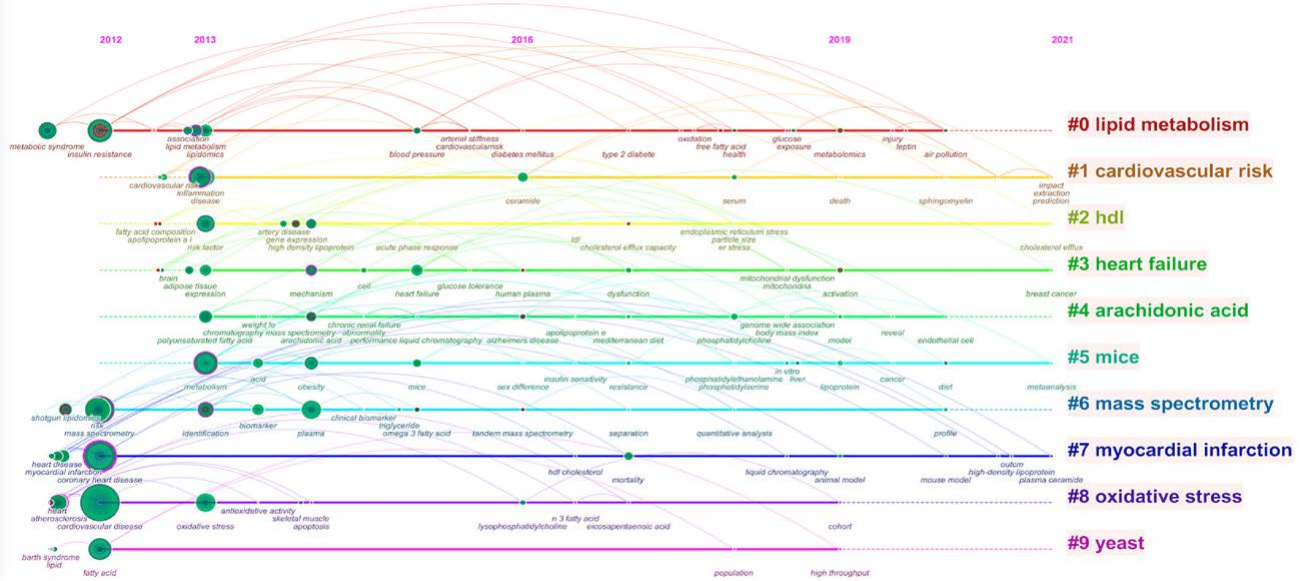
The timeline view of keywords cluster.

To further analyze the core content of all keywords comprehensively and systematically, we performed cluster analysis of the keywords using the built-in log-likelihood ratio algorithm of the CiteSpace software (Version 5.8.R3, Chaomei Chen, Drexel University). Figure 3 presents the clustering results from a timeline perspective. The smaller the value of the cluster label, the larger the size of the cluster. Silhouette *S* = 0.8853 and modularity *Q* = 0.7439 showed that the clustering structure was good, and the clustering results were highly credible. Heart failure and myocardial infarction in the cluster label reflected the main application direction of lipidomics in CVDs. High-density lipoprotein (HDL) and yeast were the focus points. Lipid-related metabolic pathways mainly include arachidonic acid and oxidative stress. Experimental studies on lipidomics often use mice as the main experimental animal.

### 3.7. Keywords burst analysis

**Figure 4.**
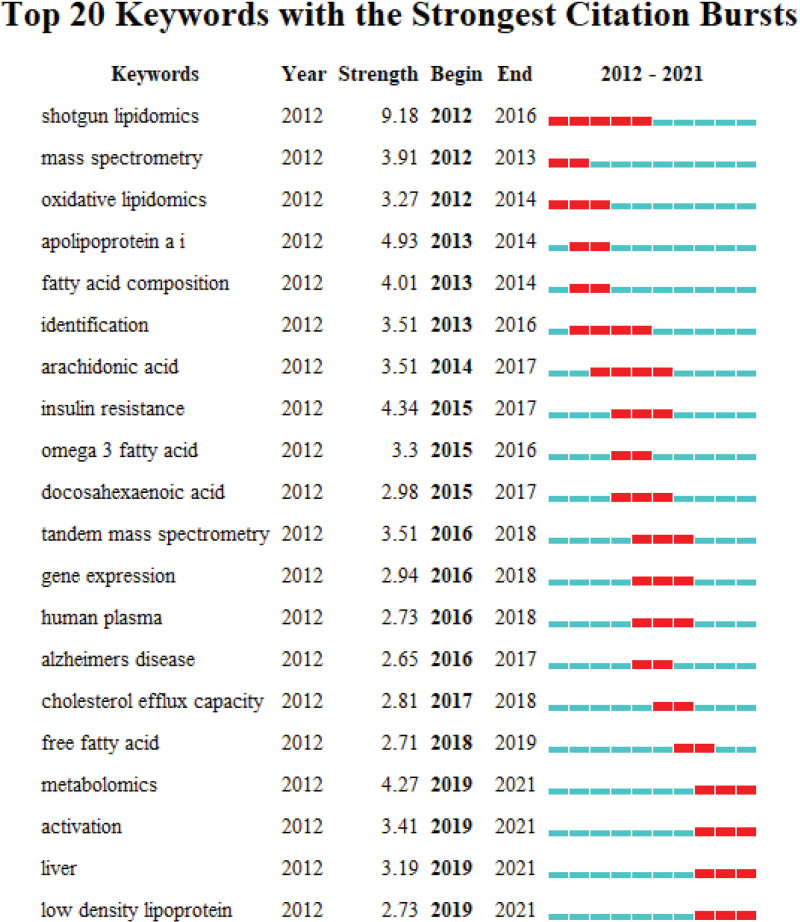
The top 20 burst keywords.

CiteSpace was further used to conduct keyword burst analysis to identify the trend of keyword development over time and to reveal the research hotspots in the field within a specific period. Similarly, current research frontiers in the field are revealed based on keywords that have emerged in recent years. In Figure 4, the timeline is depicted as a blue line, and the red square section on the blue line indicates the research hotspot of a keyword within a period. The top 20 keywords are shown in the figure according to the order of the beginning times of the keywords. The series of keywords that appeared in 2012 included shotgun lipidomics, mass spectrometry, and oxidative lipidomics. The burst intensity of shotgun lipidomics was the strongest (9.18), indicating that it lasted the longest until 2016, when the heat declined. Thereafter, identification, arachidonic acid, and docosahexaenoic acid were the hot keywords during the 2012 to 2017 period. Since then, tandem mass spectrometry, gene expression, and human plasma have been the most dominant keywords, which continued dominant for >3 years. Metabolomics, activation, liver, and low-density lipoprotein started to explode in 2019 and have continued to be the frontier of research within the last 3 years of the study period.

## 4. Discussion

Based on the number of publications per year, the application of lipidomics in CVD was in its infancy in 2012, and the number of publications has steadily increased annually since then, which may be related to the increasing number of patients worldwide. This also indicates that the importance of lipidomics in cardiovascular system diseases is receiving increasing attention from researchers. Collectively, this research field is currently in the phase of rapid development.

The USA has a greater advantage in terms of the number of publications, publication time, and BC value. China ranks 2nd in the number of publications, but not in the BC value, indicating that China is equally strong in this field, but does not have strong research collaborations with other countries. The Chinese Academy of Sciences and the University of Chinese Academy of Sciences are the leading research institutions in China, ranking 2nd and 8th in the institutional rankings, respectively. However both institutions have low BC values, indicating a need to strengthen research collaborations. It is worth noting that although Australia ranks 5th in the national rankings, the Baker Heart and Diabetes Institute, Monash University, and University of Melbourne from Australia rank 1st, 4th, and 7th, respectively in the institutional rankings, indicating that the academic influence of researchers from these institution radiates globally. Eighty percent of the top 10 countries are in Europe and North America, which may have been influenced by language and geographical factors. However, at the institutional level, the distribution of the top 10 institutions in Europe, North America, Asia, and Australia is relatively balanced, indicating that this research area has attracted the attention of researchers in most regions of the world.

Peter J Meikle from the Baker Heart and Diabetes Institute in Australia is the most prolific and co-cited author in the field, with a focus on the systems biology domain and metabolomics, and is the co-lead of the Obesity and Lipids Program.^[24]^ From 2002 to 2008, he focused on the use of plasma lipidomics to diagnose and assess the therapeutic efficacy of Gaucher disease as well as for predicting disease severity.^[25,26]^ In 2011, he measured 305 lipid species in patients with stable CAD and acute coronary syndrome (ACS) and in healthy controls using electrospray ionization tandem mass spectrometry. From this analysis, he identified a total of 50 individual lipid species significantly associated with ACS and 102 lipids significantly associated with stable CAD based on binary logistic regression analysis. These results suggest that a risk assessment model combining plasma lipidomics with traditional risk factors has the potential to better distinguish unstable CAD and it can be used as a predictor of ACS.^[27]^ In addition, Meikle conducted a plasma lipidomic analysis based on a large population cohort,^[28]^ explored the relationship of lipid species with type 2 diabetes and prodromal diabetes,^[29]^ and validated the predictive role of plasma lipid profile on the risk of cardiovascular events in type 2 diabetes through a randomized controlled clinical trial.^[30]^ All of these studies have greatly promoted the application of lipidomics in the above diseases, and the related research results have been widely cited by other scholars, which has strongly promoted the development of this field. Han and Gross pioneered the concept of shotgun lipidomics based on electrospray ionization mass spectrometry.^[31]^ Their work has greatly contributed to the development of lipidomics by enabling the identification and quantification of most major and many minor lipid classes directly from biological lipid extracts without the need for chromatographic purification and by providing rapid, highly sensitive identification of hundreds of lipids that are missed by other methods. Based on the Bruneck study, Stegeman extracted 685 plasma samples and identified 105 lipid species from 8 different lipid classes by shotgun lipidomics using a triple-quadrupole mass spectrometer.^[32]^ The study results showed that among these lipid species, triacylglycerols and cholesterol esters, as well as phosphatidylethanolamines, have the strongest predictive effect on CVD risk; therefore, it is recommended that these 3 lipid species should also be considered to improve the predictive efficacy of risk.

Six of the top 10 authors in the reference co-citation analysis were among the top 10 authors in the author co-occurrence and co-citation analyses, with representative works by Meikle and Stegeman as discussed above. The remaining references deal with the role of plasma ceramides in the prediction of mortality risk in coronary heart disease,^[33,34]^ the significance of lipidomics in predicting cardiovascular risk in type 2 diabetes, and the protective role of the HDL lipidome in atherosclerotic disease.^[35,36]^

A series of keyword-based analyses showed that CAD, heart attack, atherosclerosis, heart failure, hypertension, HDL cholesterol, apolipoprotein a, omega3 fatty acid, oxidative stress, inflammation, risk factors, and cardiovascular risk are the most relevant research hotspots for lipidomics in cardiovascular and cerebrovascular system diseases.^[37,38]^ In clinical practice, CAD, hypertension, heart failure, and arrhythmias are the most common conditions in a vasculocardiology department, and atherosclerosis is an important factor in the CAD development. We found that the application of lipidomics in CAD or atherosclerosis can be broadly divided into 2 categories by tracing the relevant primary literature. One of the 2 categories is to explore the lipid profiles of different disease subtypes, such as the characteristic lipid species of symptomatic coronary microangiopathy and acute myocardial infarction,^[37–39]^ and the lipid profiles of plaques at different stages of atherosclerosis development.^[40]^ The other is to explore the mechanisms of therapeutic effects based on lipidomic testing, such as proprotein convertase subtilisin/kexin type 9 inhibitors,^[41,42]^ lifestyle interventions such as diet structure adjustment, limitation of smoking and alcohol consumption, and the intervention of effective active ingredients in Chinese herbs.^[43–45]^ Applications in heart failure and hypertension have also focused on exploring the characteristic lipid species associated with these diseases and the predictive role of lipid profiles on disease risk and cardiovascular outcomes based on clinical trials.^[46–48]^ No lipidomic studies have been performed specifically for clinical arrhythmia, and studies that have been performed have focused on exploring the serum lipid profile of fatal arrhythmias secondary to other diseases.^[49,50]^ In addition, the keywords burst analysis suggested that metabolomics, activation, liver, and low-density lipoprotein became active keywords since 2019.

Lipid metabolism disorders can lead to a variety of clinical diseases, and the application of lipidomic technologies has expanded our understanding of the physiopathology of lipid metabolism disorders. Atherosclerosis is an important pathological basis for CAD, and lipidomics has become an important tool to discover potential lipid molecular markers for CAD. Circulating lipid profiles have been widely incorporated into risk prediction models of cardiovascular events.^[51,52]^ Simultaneously, enzymes related to lipid metabolism have emerged as promising therapeutic targets to guide the development of new drugs for coronary heart disease. Drugs can be divided into 2 main categories: receptor drugs that upregulate protective lipid molecules; and inhibitors, monoclonal antibodies, and receptor antagonists, which downregulate harmful lipid molecules.^[53–55]^ Drugs that have been developed from research include inhibitors of sphingolipid biosynthesis,^[56]^ drugs targeting sphingosine-1-phosphate,^[57]^ and drugs targeting lysophosphatidic acids.^[58]^

### 4.1. Limitations

This study has some limitations. All the literature in this study was extracted from the WoSCC database, and the language was limited to English, which may have led to literature selection bias. However, WoSCC is the most used database in the field of bibliometrics, and many bibliometric studies that used this database have been published,^[59–61]^ implying that its individual application can provide a comprehensive summary of research trends in this field.

## 5. Conclusions

The results of this study suggest that the application of lipidomics in CVDs has been fruitful over the last decade and has high research value and application prospects. However, according to the number of publications each year, this field remains a relatively new research area. The results of this study can provide a reference for researchers to quickly identify key countries, institutions, researchers, core references, and hot frontiers of research in this field. We expect that this study will further advance the application of lipidomics in the cardiovascular system and better improve clinical practice. In the coming years, more in-depth knowledge of molecular lipid species that contribute to the pathophysiology of CVD may provide better insight into biomarkers and novel therapeutic targets in cardiovascular medicine.

## Author contributions

WW designed the study. LS collected and verified the data. LS and WW performed software analyses. WW drafted the first version of this manuscript. LS revised the manuscript.

**Data curation:** Wenting Wang, Lei Song.

**Formal analysis:** Lei Song.

**Writing – review & editing:** Wenting Wang.

## Supplementary Material
