# Supplementary material for: Landscape of lipidomics in cardiovascular medicine from 2012 to 2021: A systematic bibliometric analysis and literature review
Source: Medicine (Baltimore). 2022 Dec 30;101(52):e32599. doi: 10.1097/MD.0000000000032599 (PMC9803420; doi:10.1097/MD.0000000000032599)
Supplement: Supplementary file 1 [file medi-101-e32599-s001.pdf]

Supplemental Digital Content (Table S1): The 10 countries and institutions with the highest volume of collaborative publications

Rank	Frequency	Centrality	Country	Year	Rank	Frequency	Centrality	Institution
1	269	0.12	USA	2012	1	28	0.09	Baker Heart and Diabetes Institute
2	128	0.06	PEOPLES R CHINA	2012	2	21	0.08	Chinese Academy of Sciences
3	83	0.02	GERMANY	2012	3	16	0.12	Harvard Medical School
4	78	0.38	UK	2012	4	16	0.05	Monash University
5	73	0.17	AUSTRALIA	2012	5	14	0.00	Harvard T.H. Chan School of Public Health
6	49	0.05	SPAIN	2013	6	12	0.29	Imperial College London
7	45	0.28	FRANCE	2012	7	11	0.18	University of Melbourne
8	39	0.02	CANADA	2013	8	11	0.02	University of the Chinese Academy of Sciences
9	39	0.33	ITALY	2012	9	11	0.05	Leiden University
10	38	0.37	NETHERLANDS	2012	10	11	0.02	Capital Medical University
