# Supplementary material for: Landscape of lipidomics in cardiovascular medicine from 2012 to 2021: A systematic bibliometric analysis and literature review
Source: Medicine (Baltimore). 2022 Dec 30;101(52):e32599. doi: 10.1097/MD.0000000000032599 (PMC9803420; doi:10.1097/MD.0000000000032599)
Supplement: Supplementary file 2 [file medi-101-e32599-s002.pdf]

Supplemental Digital Content (Table S2): The top 10 authors in the co-occurrence and co-citation analyses

Rank	Frequency	Centrality	Author	Year	Frequency	Centrality	Co-cited Author	Year
1	47	0.04	Peter J Meikle	2013	142	0.17	Peter J Meikle	2013
2	18	0.03	Gerard Wong	2013	122	0.15	Xianlin Han	2012
3	17	0.00	Jacquelyn M Weir	2013	116	0.19	Christin Stegeman	2013
4	14	0.05	Christopher K Barlow	2013	84	0.03	Oswald Quehenberger	2013
5	10	0.01	Kevin Huynh	2017	81	0.04	Jordi FOLCH	2012
6	9	0.01	Joanne E Curran	2013	65	0.18	E G Bligh	2012
7	9	0.01	John Blangero	2013	62	0.09	Reijo Laaksonen	2017
8	9	0.01	Piyushkumar A Mundra	2016	60	0.01	Yoav Benjamini	2013
9	9	0.00	Anatol Kontush	2015	58	0.45	Eoin Fahy	2013
10	7	0.00	Corey Giles	2018	52	0.37	Eugene P Rhee	2013
