# Supplementary material for: Landscape of lipidomics in cardiovascular medicine from 2012 to 2021: A systematic bibliometric analysis and literature review
Source: Medicine (Baltimore). 2022 Dec 30;101(52):e32599. doi: 10.1097/MD.0000000000032599 (PMC9803420; doi:10.1097/MD.0000000000032599)
Supplement: Supplementary file 3 [file medi-101-e32599-s003.pdf]

Supplemental Digital Content (Table S3): The top 10 journals and co-cited journal

Rank	Journal	Count	Impact Factor (2020)	JCR	Co-cited Journal	Co-cited frequency	Impact Factor (2020)	JCR
1	JOURNAL OF LIPID RESEARCH	30	5.922	Q1	JOURNAL OF LIPID RESEARCH	535	5.922	Q1
2	ATHEROSCLEROSIS	21	5.162	Q2	PLoS ONE	477	3.240	Q2
3	METABOLITES	21	4.932	Q2	CIRCULATION	450	29.690	Q1
4	PLOS ONE	21	3.240	Q2	JOURNAL OF BIOLOGICAL CHEMISTRY	428	5.157	Q2
5	JOURNAL OF PROTEOME RESEARCH	19	4.466	Q1	ARTERIOSCLEROSIS THROMBOSIS AND VASCULAR BIOLOGY	326	8.313	Q1
6	INTERNATIONAL JOURNAL OF MOLECULAR SCIENCES	18	5.924	Q1	PROCEEDINGS OF THE NATIONAL ACADEMY OF SCIENCES OF THE UNITED STATES OF AME	309	10.700	Q1

7	SCIENTIFIC REPORTS	18	4.380	Q1	BIOCHIMICA ET BIOPHYSICA ACTA-MOLECULAR AND CELL BIOLOGY OF LIPIDS	306	4.698	Q2
8	BIOCHIMICA ET BIOPHYSICA ACTA-MOLECULAR AND CELL BIOLOGY OF LIPIDS	12	4.698	Q2	JOURNAL OF CLINICAL INVESTIGATION	297	14.808	Q1
9	MOLECULAR NUTRITION & FOOD RESEARCH	12	5.820	Q1	NEW ENGLAND JOURNAL OF MEDICINE	287	91.253	Q1
10	LIPIDS IN HEALTH AND DISEASE	11	3.876	Q3	ATHEROSCLEROSIS	284	5.162	Q2
