# Supplementary material for: Landscape of lipidomics in cardiovascular medicine from 2012 to 2021: A systematic bibliometric analysis and literature review
Source: Medicine (Baltimore). 2022 Dec 30;101(52):e32599. doi: 10.1097/MD.0000000000032599 (PMC9803420; doi:10.1097/MD.0000000000032599)
Supplement: Supplementary file 4 [file medi-101-e32599-s004.pdf]

Supplemental Digital Content (Table S4): The top 20 keywords

Rank	Frequency	Centrality	Keyword	Rank	Frequency	Centrality	Keyword
1	210	0.18	cardiovascular disease	11	59	0.28	inflammation
2	126	0.41	coronary heart disease	12	56	0.18	risk factor
3	97	0.25	mass spectrometry	13	51	0.10	metabolic syndrome
4	93	0.02	risk	14	49	0.47	atherosclerosis
5	87	0.28	metabolism	15	46	0.11	cholesterol
6	83	0.17	fatty acid	16	43	0.07	heart
7	78	0.16	insulin resistance	17	42	0.38	identification
8	65	0.00	oxidative stress	18	41	0.07	myocardial infarction
9	65	0.04	disease	19	38	0.10	shotgun lipidomics
10	62	0.05	plasma	20	36	0.14	obesity
